# Attachment, Entry, and Intracellular Trafficking of Classical Swine Fever Virus

**DOI:** 10.3390/v15091870

**Published:** 2023-09-03

**Authors:** Xin Guo, Maolin Zhang, Xiaomin Liu, Yannan Zhang, Chongyang Wang, Yidi Guo

**Affiliations:** State Key Laboratory for Diagnosis and Treatment of Severe Zoonotic Infectious Diseases, Key Laboratory for Zoonosis Research of the Ministry of Education, Institute of Zoonosis, College of Veterinary Medicine, Jilin University, Changchun 130012, China

**Keywords:** classical swine fever virus, attachment, entry, intracellular trafficking

## Abstract

Classical swine fever virus (CSFV), which is a positive-sense, single-stranded RNA virus with an envelope, is a member of the *Pestivirus* genus in the *Flaviviridae* family. CSFV causes a severe and highly contagious disease in pigs and is prevalent worldwide, threatening the pig farming industry. The detailed mechanisms of the CSFV life cycle have been reported, but are still limited. Some receptors and attachment factors of CSFV, including heparan sulfate (HS), laminin receptor (LamR), complement regulatory protein (CD46), MER tyrosine kinase (MERTK), disintegrin, and metalloproteinase domain-containing protein 17 (ADAM17), were identified. After attachment, CSFV internalizes via clathrin-mediated endocytosis (CME) and/or caveolae/raft-dependent endocytosis (CavME). After internalization, CSFV moves to early and late endosomes before uncoating. During this period, intracellular trafficking of CSFV relies on components of the endosomal sorting complex required for transport (ESCRT) and Rab proteins in the endosome dynamics, with a dependence on the cytoskeleton network. This review summarizes the data on the mechanisms of CSFV attachment, internalization pathways, and intracellular trafficking, and provides a general view of the early events in the CSFV life cycle.

## 1. Introduction

Classical swine fever virus (CSFV) is a member of the *Pestivirus* genus within the *Flaviviridae* family. Classical swine fever (CSF), which is caused by the virus, was first recognized in 1833 in Ohio, USA, and the virus was identified in 1904 [1]. CSFV is highly infectious and is identified as a notifiable disease by the World Organization for Animal Health, posing a threat to the worldwide pig farming industry [2]. Disease symptoms include high fever, leukopenia, abortion, and hemorrhaging of the skin and internal organs, along with neurological symptoms, resulting in high mortality [3]. CSFV can be transmitted via direct contact between animals or indirect contact through infectious secretions, such as contaminated food or semen [4]. Transmission can also occur vertically by crossing the placenta. The virus invades the host through the mucous membranes of the oral and nasal cavities, initiates production in the tonsils, and then spreads all over the lymphatic system via the blood. CSFV tends to reproduce in blood, lymphoid tissue, the ileum, and pancreas, which are preferred sites of acute infection, while CSFV is unlikely to replicate in the brain, heart, and duodenum [5]. CSFV has a distinct tropism toward cells of the immune system, such as T cells, B cells, and monocytes, leading to severe cell necrosis and lysis, which is closely associated with pathological features [6,7].

In this review, we discuss the strategies implemented by CSFV to accomplish early intracellular events, including attachment, internalization, and trafficking. This can help in understanding the biology of the virus and highlighting potential mechanisms in the life cycle of CSFV.

## 2. Structure of CSFV and Viral Encoded Proteins

**Figure 1:**
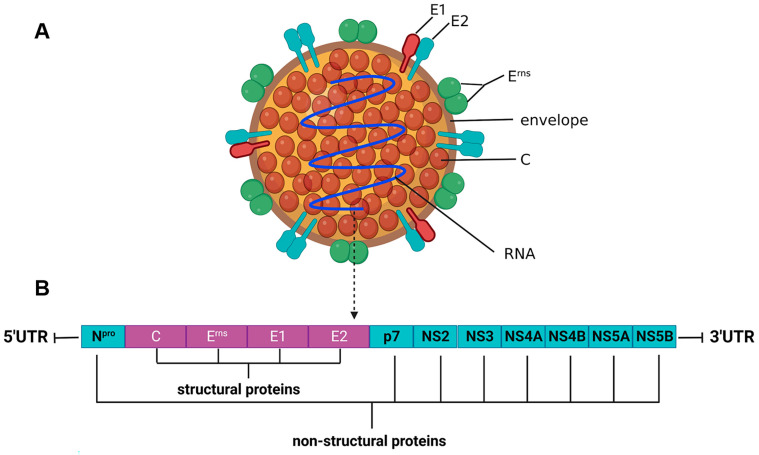
Basic features of CSFV and the organization of CSFV genome. (**A**) Schematic outline of a CSFV particle. CSFV is an enveloped, single-stranded, and positive-sense RNA virus. Three envelope proteins of CSFV-E^rns^, E1, and E2 are located on the external part of viral particles. As transmembrane proteins, E2 is present as homodimers or forms E1–E2 heterodimers on the virion. Unlike E1 buried underneath, E^rns^ is exposed on the outside surface of the envelope with E2 and exists as a dimer. C proteins condense viral RNA genome with an unspecific affinity for nucleic acids. (**B**) Schematic organization of CSFV genome. The length of the genome is approximately 12.3 kb. The viral genome is composed of a large open reading frame (ORF), a 5’-noncoding region (5′-UTR), and a 3’-noncoding region (3’-UTR). The ORF encodes a precursor polyprotein, which is cleaved into four structural proteins (C, E^rns^, E1, and E2) and eight nonstructural proteins (N^pro^, p7, NS2, NS3, NS4A, NS4B, NS5A, and NS5B).

CSFV is an enveloped, single-stranded, positive-sense RNA virus. The length of the genome is approximately 12.3 kb. The viral genome is composed of a large open reading frame (ORF), a 5’-noncoding region (5′-UTR), and a 3’-noncoding region (3’-UTR). The ORF encodes a precursor polyprotein comprising 3898 amino acids (aa). The single, large polyprotein is cleaved by cellular and viral proteases into 12 proteins, including eight nonstructural proteins (N^pro^, p7, NS2, NS3, NS4A, NS4B, NS5A, and NS5B) and four structural proteins (C, E^rns^, E1, and E2) [3,8] (Figure 1).

### 2.1. Structural Proteins of CSFV

The capsid protein (C) is flexible in length and differs across *Pestivirus* species, which could condense viral RNA genome with an unspecific affinity for nucleic acids [9]. It was reported that CSFV lacking nearly the entire core coding region is still viable upon the introduction of a single amino-acid substitution (SAAS; N_2256_Y) in the helicase domain of NS3, suggesting that the C protein is dispensable in CSFV propagation in vitro due to the compensatory function of the NS3 protein [10]. E^rns^, which consists of 227 amino acids with a molecular weight of about 26 kDa, directly follows the C protein. Generally, E^rns^ forms a homodimer on the virion [11,12]. It appears in a membrane-bound form with a long amphipathic helix at its C-terminus anchored into the membrane [13], and it possesses unique ribonuclease activity critical for viral replication [14,15,16]. The interaction between E^rns^ and membrane-associated heparan sulfate (HS), laminin receptor (LamR), or complement regulatory protein (CD46) mediates CSFV attachment to host cells [17,18,19,20]. In addition, cell experiments showed that E^rns^ is able to antagonize type I interferon (IFN) signaling [21,22]. E1 is a membrane glycoprotein consisting of 195 aa, with an N-terminal extracellular domain and a C-terminal hydrophobic transmembrane segment [23], as well as several cysteine residues [24]. E1 forms disulfide-linked heterodimers with E2 on the viral particle, and E1–E2 heterodimers mediate viral endocytosis [25,26]. E2 is a glycoprotein of approximately 55 kDa that consists of 373 amino acids [23]. It also presents as a homodimer. It belongs to transmembrane protein Ⅰ, and it possesses an N-terminal signal peptide and a hydrophobic transmembrane segment in the C terminus region that anchors the E2–E1 and E2–E2 dimers in the viral lipid membrane [11]. E2 accounts for the species tropism of pestiviruses [27] and is the most immunogenic of the glycoproteins due to its induction of neutralizing antibodies [23].

### 2.2. Nonstructural Proteins of CSFV

N^pro^, which is 168 aa in length, demonstrates autoproteolytic activity, with its N-terminal protease operating the cleavage between the N^pro^ and C proteins [8]. N^pro^ plays a role in restraining host cell apoptosis and blocking the cellular IFN response during virus infection [28,29,30,31,32,33]. As a metalloprotein, it contains a conserved metal-binding TRASH motif, which coordinates zinc ions via Cys112, Cys134, and Cys138, and it is important for protein stability and N^pro^-mediated IRF-3 degradation [34]. P7, which is encoded downstream of E2, is a small hydrophobic polypeptide with a molecular mass of 6–7 kDa. It is a pore-forming protein, with its pore-forming activity positioned in the C-terminal transmembrane helix, and it plays a role in viral virulence and budding. Reverse genetics experiments confirmed that recombinant CSFVs with p7 gene mutation were partially or completely attenuated in swine relative to the highly virulent strain [35,36].

NS2, which consists of about 450 amino acids, is also a transmembrane protein with auto-protease activity. The active site of the NS2 protease is composed of a catalytic triad of His1447, Glu1462, and Cys1512 [37]. NS2 protease only plays a role in NS2–NS3 cleavage in the *cis* conformation, and the NS2 C-terminus stays in the active site of the protease after *cis*-cleavage to prevent further cleavage in the *trans* formation [38,39]. Previous papers reported that uncleaved NS2–NS3 is crucial for the generation of infectious virion [40]. NS3 (about 80 kDa) is a multifunctional protein with a chymotrypsin-like serine protease that resides in the N-terminal domain [41,42] and a helicase and NTPase domain that are located in the C-terminal domain [43].

NS4A has a molecular weight of about 10 kDa and is composed of a hydrophobic membrane spanning the N-terminal region and a C-terminal cytosolic domain [44]. It acts as a cofactor of NS3 protease and is indispensable in virion morphogenesis, RNA replication, and viral replicase assembly [40,45]. NS4B is a hydrophobic protein with a size of around 35 kDa and is localized at intracellular membranes [46]. NS4B encompasses two conserved domains: the Walker A motif (209–216 aa), which exhibits NTPase activity, and the Walker B motif (335–342 aa) [47]. NS4B expressed by bacteria hydrolyzes GTP and ATP, and this activity was found to be inhibited when mutations were introduced into the two conserved domains [48].

NS5A (58 kDa) is highly phosphorylated by cellular kinases, similar to NS5 of the Flavivirus genus [49]. An amphipathic α-helix at its N-terminus is anchored to intracellular membranes. It was shown to be involved in viral RNA replication by binding to NS5B and 3′UTR, which regulates viral RNA synthesis [50,51]. In addition, NS5A has a negative regulatory effect on the IRES-mediated translation of reporter mRNA [52]. NS5B, with a size of about 77 kDa, is an RNA-dependent RNA polymerase (RdRp) that shows polymerase activity in vitro. Enhancing RNA synthesis using high GTP concentrations was found to facilitate de novo origination of RNA biosynthesis or generate copy-back products [53,54,55,56]. NS5B can also suppress the inhibitory effect of NS5A on viral translation by binding to NS5A [57].

## 3. Attachment to Host Cells Is Dependent on Receptors

Attachment to host cells initiates the life cycle of the virus and determines the viral tropism. The receptor-mediated endocytosis of CSFV is thought to be mediated by Erns and E2 proteins, which bind to particular receptors and allow viruses to access host cells [26,58]. E1 and E2 are both class II fusion proteins with internal fusion peptides. E2 has a truncated transmembrane domain that causes fusion to be blocked in hemifusion [24,59,60,61], and E1 only has half the size of typical class II fusion proteins [62]. It is speculated that E1 must span the distance to the membrane to mediate fusion, possibly using E2 as a scaffold. A hypothesis of CSFV early infection is proposed: first, Erns mediates the contact between virus and host cells; second, E2 attaches to particular surface molecules; third, E1 and E2 cooperate through the fusion loop of E1 and the stem of E2, promote fusion with the membrane of the host cell, and then penetration is induced [25,58,63]. 

To date, HS, LamR, and CD46 have been definitively identified as attachment receptors of CSFV by recognizing Erns [17,18,19,20]. Meanwhile, cellular surface proteins, like MER tyrosine kinase (MERTK), disintegrin, and metalloproteinase domain-containing protein 17 (ADAM17), were reported to be attachment factors assisting in viral endocytosis by binding with E1–E2 heterodimers [64,65]. The roles of the above involved factors are listed in Table 1. Furthermore, the SE24 peptide of the E2 protein (amino acids 955–1004) was identified to be critical in the attachment to porcine kidney-15 (PK-15) cells [66]. 

### 3.1. HS

Glycoaminoglycans (GAGs) are unbranched polysaccharide chains composed of repeating disaccharide sequences. Common GAGs include chondroitin sulfate (CS), dermatan sulfate (DS), keratan sulfate (KS), heparan sulfate (HS), heparin, and hyaluronic acid (HA). HS is present on the surface of virtually all types of cells and in the extracellular matrix of most animal tissues, functioning in development and homeostasis [69]. Various pathogens were reported to hijack HS for use as attachment factors, including herpes simplex virus (HSV) [70], human immunodeficiency virus type 1 (HIV) [71,72], and foot-and-mouth disease virus (FMDV) [73].

Hulst et al. first proposed HS as a CSFV E^rns^ receptor. The most common GAGs, namely, chondroitin sulfate (CS) A, B, and C, as well as HS, were tested to determine whether they could mediate the initial binding of CSFV to cells. By employing Brescia clone 1.1.1 (C1.1.1), which is a CSFV strain obtained via an extensive passage in swine kidney (SK6) cells, heparin (a higher sulfated HS structural analog) and dextran sulfate (an artificial highly sulfated polysaccharide) were found to impede viral infection, while HS and CS A, B, and C had no effect. Removing HS from the surface of SK6 cells through heparinase I treatment almost completely abolished viral intracellular infection. The heparin HS-type polysaccharide chain without additional cell surface molecules is sufficient to immobilize CSFV virus particles. C1.1.1 virus particles and CSFV E^rns^ purified from insect cells attached to immobilized heparin, while purified E2 did not, which suggests that E^rns^, but not E2, has the ability to bind HS. Interestingly, infection with viral clones obtained from infected pig blood or minimal cell passage was not influenced by heparinase I or heparin. However, following a second round of amplification in SK6 cells, these viral clones reacquired their sensitivity to drug treatment. Sequencing analysis revealed that a key residue at the C terminus of E^rns^ (amino acid 476 in the polyprotein) determines the affinity for HS. The substitution of a positively charged Arg residue for a neutral Ser residue after a passage in SK6 cells is sufficient for the occupation of HS as an E^rns^ receptor [18].

The role of HS as a receptor in facilitating CSFV binding was further validated in vivo. Considering the essential role of amino acid 476 of E^rns^ in affinity with HS, an HS-independent recombinant virus (S-ST virus) with Ser476 and an HS-dependent recombinant virus (S-RT virus) with Arg476 were constructed using reverse genetics. After determining that the adaptive Ser-to-Arg mutation had no impact on CSFV pathogenicity, these recombinant viruses reisolated from pigs were analyzed. In only one of three pigs infected with the S-RT strain, the S-RT virus converted into an S-ST strain with Arg476 mutating back to Ser476, which reduced immobilization for HS. The virus reisolated from pigs infected with the S-ST virus could infect cultured and primary swine kidney cells via an HS-dependent mechanism. However, this in vivo generation introduced no mutations in the genes of viral envelope proteins E^rns^, E1, and E2 according to a sequence analysis. This indicates that although CSFV possesses Ser476 in the C terminus of E^rns^, the surface properties of CSFV generated in pigs vary from those of genetically identical viruses produced in SK6 cell culture, and there is a high affinity for the HS receptor. CSFV can still infect SK6 cells after a single amino-acid mutation in the E^rns^ protein, which abrogates the ability to attach HS and the broad distribution of HS does not explain the restricted host range of CSFV. Moreover, heparin and heparinase I treatment had no effect on lung macrophages infected with the S-ST virus generated in vivo or even the HS-dependent S-RT virus variant. Hence, in addition to HS, alternative receptors are involved in the attachment of CSFV to cells [17].

### 3.2. LamR

LamR is a cell surface receptor for laminin. It functions via the adhesion of cells to the basement membrane and in the consequent activation of signaling transduction pathways. It also plays a role in cell fate determination and tissue morphogenesis, and it acts as an attachment factor for various pathogens, including tick-borne encephalitis virus (TBEV) [74]; Sindbis virus (SINV) [75]; Venezuelan equine encephalitis virus (VEEV) [76]; dengue virus (DENV) [77,78,79,80]; and adeno-associated virus (AAV) serotypes 8, 2, 3, and 9 [81].

Chen et al. identified LamR as another CSFV cell receptor. By employing a set of small interfering RNAs (siRNAs) against several porcine cell membrane protein genes, LamR knockdown significantly reduced CSFV titers. Coimmunoprecipitation (co-IP) assays revealed that the CSFV envelope protein E^rns^ interacts with LamR, and confocal analysis showed that LamR is colocalized with CSFV virions on the membrane. Anti-LamR antibodies, laminin, and soluble LamR were found to impede CSFV infection in a dose-dependent manner. Higher viral titers were observed when recombinant lentiviruses expressing and overexpressing LamR were both transduced into PK-15 cells. Attachment experiments showed that, while the interaction of LamR with CSFV allowed for CSFV infection, LamR expression could not confer susceptibility to CSFV on nonpermissive cells, which indicates that LamR acts as an attachment factor but is not involved in entry. Moreover, LamR functions as a substitute attachment receptor with a supplementary role in HS, especially in SK6 cells [19].

### 3.3. CD46

Complement regulatory protein CD46 is a widely expressed complement regulatory protein and a cofactor for serine protease factor I to cleave and inactivate C3b and C4b deposited on the cell [82]. CD46 was identified as a receptor for measles virus (MeV) vaccine strains, human herpesvirus 6 (HHV-6), adenovirus (AdV), etc [83]. It is also regarded as the main cellular receptor for bovine viral diarrhea virus (BVDV) [84,85]. CD46_bov_ is an important entry factor for the HoBiPeV strain HaVi-20 but not for the GPeV strain PG-2 of BVDV, suggesting that some strains of BVDV are dependent on CD46 and others are not [86]. 

Dräger et al. found that there was a reproducible inhibition of CSFV growth of monoclonal antibodies (Mabs) against porcine CD46 [20]. However, infection of CRISPR/Cas-generated CD46 knockout cells based on different kidney epithelial cells (slow phase eye velocity (SPEV) and PK-15 cells) and 38A_1_D lymphoma cells, which are naturally deficient for CD46, confirmed the CD46-independent entry, illustrating that CD46 is not an essential determinant of host cell entry for CSFV [67].

### 3.4. MERTK

MERTK is a tyrosine kinase receptor that belongs to the TAM (TYRO3, AXL, and MERTK) receptor family. It regulates many physiological processes, including cell survival, migration, and differentiation, as well as phagocytosis of apoptotic cells. MERTK transduces downstream signals through the phosphorylation of MAPK1, MAPK2, FAK/PTK2, or RAC1, and it functions by inhibiting the Toll-like receptor (TLR)-mediated innate immune response [87,88]. Many TAM receptors were reported to potentiate infection by various viruses. For example, AXL facilitates entry by Zaire ebolavirus (ZEBOV) [89], Simian virus 40 (SV40) [90], and Zika virus (ZIKV) [91,92,93], while TYRO3 can mediate DENV endocytosis via the clathrin-dependent pathway [94,95,96]. Little was known about the role of MERTK in viral infections until Zheng et al. revealed that MERTK is a CSFV attachment factor [64].

Transcriptomic analysis and RNA interference screening were performed to screen out MERTK as a candidate protein involved in CSFV infection. CSFV replication dramatically decreases in MERTK-knockdown cells. Anti-MERTK and soluble MERTK ectodomains were found to impair CSFV genome copies and progeny viral titers in a dose-dependent manner. Co-IP confirmed the interaction between MERTK and CSFV E2 protein. According to RT-qPCR data, anti-MERTK or soluble MERTK ectodomain treatment and MERTK knockdown interfered with CSFV entry into PK-15 cells, illustrating that MERTK can facilitate CSFV entry. Moreover, the antagonistic effect of MERTK on the host’s innate immune response was verified by quantifying the mRNA levels of IFN-β and suppressor of cytokine signaling protein 1 (SOCS1) in MERTK-knockdown cells infected with CSFV. These findings indicate that MERTK facilitates CSFV entry while also acting as a potent anti-CSFV interferon-stimulated gene (ISG) [64,97,98].

### 3.5. ADAM17

ADAM17, also named tumor-necrosis-factor-α-converting enzyme (TACE), is a member of the metalloproteinase superfamily. It is widely expressed in almost every tissue, with very high levels in the heart, placenta, skeletal muscle, pancreas, spleen, thymus, prostate, testis, ovary, and small intestine. It is in charge of the cleavage of various transmembrane proteins, including the membrane-bound precursor of tumor necrosis factor-α (TNF-α) [99] and IL-6 receptor (IL-6R) [100]. ADAM17 is also involved in the ectodomain release of transforming growth factor-α (TGF-α), p75 TNF receptor (p75 TNFR), L-selectin [101], and β-amyloid precursor protein (βAPP) [102].

ADAM17 knockout (KO) was found to block CSFV entry in PK-15 cells, which could be fully rescued by compensating for ADAM17 via the stable expression of pig ADAM17 (pADAM17). Moreover, an ADAM17 mutant without the intracellular domain fully restores CSFV pseudoparticle (CSFVpp) infectivity in ADAM17-KO cells, suggesting the decrease of viral internalization is caused by a reduction in CSFV attachment. Human and mouse ADAM17, which are highly homologous to pig ADAM17, also confer permissiveness to CSFV. However, other ADAMs are unrelated to CSFV entry. When ADAM17 was knocked out, fewer virions with localization on the plasma membrane were visualized, and viral internalization into host cells was restrained. Silencing of TIMP-3, which is a native tissue inhibitor of ADAM17, enhanced cell susceptibility to CSFV. The metalloproteinase domain of ADAM17 directly interacts with the extracellular domain of CSFV E2, in which the 301–345 aa region of ADAM17 engages most. Aderbasib (INCB007839), which is a selective ADAM17 inhibitor currently used in clinical trials for cancer, abolished the binding of ADAM17 to E2 in a dose-dependent manner. Although the expression pattern of ADAM17 correlates with the broad tissue tropism of CSFV in pigs, it is insufficient to define ADAM17 as a determinant of the species’ host range. The above findings suggest that ADAM-17 is an attachment factor for CSFV endocytosis [65].

In addition to the above receptors and attachment factors, several cellular membrane proteins have been found to facilitate CSFV entry. Cellular membrane protein annexin 2 (Anx2), a Ca^2+^-dependent cytosolic protein associated with cholesterol [103], was upregulated in PK-15 cells following CSFV infection. The interaction of Anx2 with E2 was observed using co-IP, mass spectrometry, Western blotting, and confocal laser scanning microscopy. In the E2-expressing PK-15 cell line, the upregulation of Anx2 was observed, indicating that E2 promotes Anx2 expression. The knockdown and overexpression of Anx2 in PK-15 cells inhibited and increased CSFV replication and proliferation, respectively. Remarkably, the treatment of PK-15 cells with Anx2-specific polyclonal antibody prior to virus infection inhibited CSFV multiplication. These results indicated that Anx2 is an attachment factor that was shown to interact with CSFV E2 and promote CSFV multiplication [68]. Chen et al. reported low-density lipoprotein receptor (LDLR) knockdown reduced CSFV development [19]. Given that LDLR is involved in the entrance of some flaviviruses, including HCV [104,105], it may potentially be a molecule that induces CSFV entry. However, the anti-LDLR monoclonal antibody failed to restrict BVDV infection [106]; thus, the role of LDLR in CSFV attachment still needs further exploration. In brief, the factors mentioned here have a crucial impact on CSFV entry and invasion; however, eliminating any one of them does not entirely eradicate viral infection. The specific functions of these proteins in CSFV invasion need further verification.

## 4. Internalization Pathways

**Figure 2:**
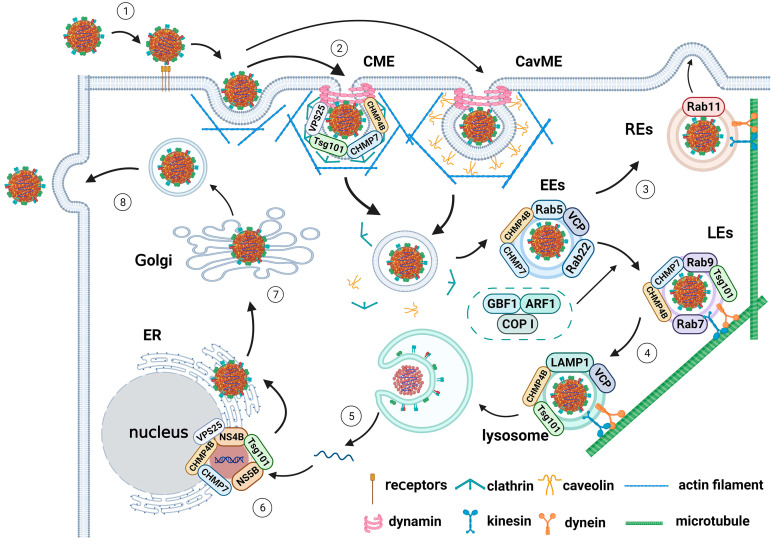
Model of CSFV entry and intracellular transport. The attachment to receptors (HS, LamR, CD46, MERTK, ADAM17, etc.) mediated by CSFV E^rns^ or E2 facilitates penetration and entry ① The CSFV particles are endocytosed within clathrin-coated endosomes (in PK-15 cells) or internalized via a caveolae-dependent pathway (in 3D4/21 cells), followed by dynamin-mediated scission from the plasma membrane, during which actin cytoskeleton is required and ESCRT proteins (Tsg101, VPS25, CHMP4B, CHMP7, etc.) are involved. Clathrin interacts with Tsg101, CHMP4B, VPS25, and CHMP7 during CSFV entry, and is then transported to the EEs ②. Subsequently, CSFV transports from EEs to LEs along microfilaments with the assistance of Rabs, ESCRT proteins (Tsg101, CHMP4B, CHMP7), and other factors (GBF1, ARF1, COP I, VCP, etc.). Among them, Rab5 and Rab7 function in coordinating the transition from early to late endosomes. Rab9 participates in the migration of cargo between late endosomes and the trans-Golgi network, and Rab11 guides the transit to REs. In the EEs, VCP/CHMP4B/CHMP7 interact with Rab5 and assist in CSFV transportation to the LEs ③. In the LEs, Tsg101/CHMP7/CHMP4B interact with Rab9 and cooperate in CSFV transportation to the lysosomes. COP I, together with GBF1 and ARF1, facilitates CSFV entry and regulates virion transport from early to late endosomes ④. In the lysosomes, the CSFV envelope fuses with the endosomal membrane for uncoating and nucleic acid releasement, with the assistance of Tsg101 and CHMP4B ⑤. In the ER lumens, ESCRT proteins interact with CSFV nonstructural proteins (NS4B/5B) forming the virus replication complex (VRC) for viral genome replication ⑥. Finally, transfer to the Golgi proceeds for processing and assembly before virion budding from the host cells ⑦ and ⑧.

Internalization, which is a normal physiological activity of cells, is the process of transferring extracellular cargo into cells through the ameboid movement of cell membranes. However, most viruses exploit cell-inherent mechanisms to complete their viral cycle. Virus internalization is a determinant process of viral infectivity and tropism. Following attachment to cell receptors, the tight association of virus particles with the host cells induces viral internalization, and then the virus enters into endosomes or other organelles. Viruses mainly enter host cells through several endocytic pathways, including clathrin-mediated endocytosis (CME), caveolae/raft-dependent endocytosis (CavME), and macropinocytosis [107]. Previous studies revealed that CSFV particles utilize the CME and CavME internalization pathways to invade host cells (Figure 2) [108,109,110].

### 4.1. Clathrin-Mediated Endocytosis of CSFV

CME is initiated by the clustering of endocytic coat proteins on the inner leaflet of the plasma membrane. The coat proteins assemble and continually recruit other cargo molecules to the coated region. Membrane bending is promoted, during which the flat plasma membrane is transformed into clathrin-coated pits (CCPs). The scission process, operated by dynamin, constricts and cuts the neck of the membrane invagination to achieve the separation of clathrin-coated vesicles (CCVs) from the plasma membrane. Actin polymerization collaborates with the coat and scission proteins to boost membrane shaping. Finally, uncoating occurs, which disassembles the pinocytic protein machinery, releasing the nascent cargo-filled vesicle and allowing for further intracellular trafficking [111]. Many enveloped viruses were reported to employ CME, including vesicular stomatitis virus (VSV) [112], infectious hematopoietic necrosis virus (IHNV) [113], rabies virus (RABV) [114,115], and BVDV [116].

The specific mechanism of CSFV uptake was not well characterized until 2016. Shi et al. proposed that CSFV internalizes within PK-15 cells through dynamin- and cholesterol-dependent and clathrin-mediated endocytosis pathways, which requires a low-pH environment and the assistance of the small GTPases Rab5 and Rab7. CSFV infection was found to be impeded by chlorpromazine (a pharmacological inhibitor of clathrin lattice polymerization) in a dose-dependent manner. EPS15 protein (an adaptor for clathrin-mediated endocytosis) and its dominant negative (DN) form (an EPS15-dominant negative mutant) were transfected into PK-15 cells. The CSFV RNA copy numbers and the percentage of CSFV E2-expressing cells were obviously reduced in the DN mutant EPS15-transfected cells compared with the control group. Silencing clathrin heavy chain (CHC) significantly suppressed CSFV RNA levels in the cells or medium, as well as the number of infected cells. These findings support the assumption that clathrin is necessary for CSFV endocytosis. Meanwhile, transfection with caveolin DN constructs and siRNA targeting caveolin-1 (siCav) did not lead to any significant reduction in viral infection, implying that CSFV invasion into PK-15 cells is independent of caveolae. Endosomal acidification is considered to be frequently associated with CME [117]. Treatment with two lysosomotropic agents, namely, chloroquine and NH_4_Cl, also inhibited CSFV infection in a dose-dependent manner. Dynasore, which is a GTPase inhibitor of dynamin activity that is required for clathrin- and caveola-mediated endocytosis, reduced the CSFV RNA copy numbers in PK-15 cells [118,119,120,121,122,123]. In addition, the viral entry decreased when cells were transfected with the DN mutant dynamin II (K44A) compared with the WT construct [108].

Cholesterol is the major component of lipid rafts (cholesterol-enriched domains) and is critical in signal transduction, protein trafficking, and other processes [124,125,126]. Numerous viruses, including African swine fever virus (ASFV) and porcine reproductive and respiratory syndrome virus (PRRSV), are involved with cellular cholesterol during their life cycle [127,128]. Using methyl-β-cyclodextrin (MβCD), which can disrupt the lipid raft by selectively extracting the membrane cholesterol, indirect immunofluorescence, and RT-qPCR assays established that the depletion of cellular membrane cholesterol influenced CSFV entry, as well as virus egress [108]. Yu et al. further confirmed the involvement of cholesterol in CSFV infection. Pretreatment of PK-15 cells with MβCD downregulated E2 protein expression, and CSFV infection was restored by supplementation with exogenous cholesterol. Attachment and internalization assays showed that cholesterol depletion had no effect on CSFV binding but influenced CSFV internalization [129].

### 4.2. Caveola-Dependent Internalization

Caveolae, which are distributed on the plasma membrane, have a size of about 50–100 nm and are an important type of raft structure. They are mainly composed of lipids (cholesterol, sphingolipid, glycosphingolipid, etc.) and proteins (caveolin, flotillin, etc.). Caveolins, including caveolin-1, -2, and -3 (CAV1, CAV2, and CAV3), are marker proteins of caveolae [130,131]. The clathrin-independent caveola- and raft-dependent pathways rely on dynamin function and are sensitive to cholesterol depletion. Dynamin GTPase activity contributes to the budding of caveolae from purified endothelial plasma membranes [132]. The association of caveolin-1 with certain raft domains invokes the invagination and budding of the vesicular structure of caveolae [133]. Caveolae are also vehicles for the invasion of diverse viruses into host cells [134,135], including foot-and-mouth disease virus (FMDV) [136], human adenovirus species D type 37 (HAdV-D37) [137], SV40, Japanese B encephalitis virus (JEV) [120,138], and Rift Valley fever virus (RVFV) [139].

Ning et al. first proposed caveola-dependent endocytosis as a new avenue of CSFV entry in porcine alveolar macrophages (3D4/21 cells), in addition to the CME pathway. Digital gene expression (DGE) analysis identified that CAV1 was significantly upregulated in 3D4/21 cells inoculated with the CSFV Shimen strain compared with mock-infected cells. CAV1 expression was increased via CSFV stimulation according to Western blotting. Images of the temporal colocalization of CSFV E2 protein and CAV1 were captured. Overexpression and silencing of CAV1 respectively promoted and reduced CSFV proliferation, which implies that caveolae play an essential role in the CSFV entry process [110]. Following transfection with the caveolin DN construct or siCav, the viral RNA copies and viral titers decreased significantly. Virions were found to be colocalized with CAV1, while there was no colocalization between virions and clathrin observed by confocal microscopy. Inconsistent with the findings in PK-15 cells [108], chlorpromazine (CPZ, which is an inhibitor of clathrin lattice polymerization) and si-CHC had no effect on the CSFV entry or binding in 3D4/21 cells. Meanwhile, wortmannin, which is an inhibitor of phosphatidylinositol 3-kinase (PI3K) [120], and 5-(N-ethyl-N-isopropyl) amiloride (EIPA), which is a micropinocytosis inhibitor, did not affect the CSFV binding or entry efficiency, suggesting that micropinocytosis was not involved. Moreover, when cells were treated with chloroquine, bafilomycin A1 (BafA1, which is an inhibitor of V-ATPase or a specific inhibitor of acidification of endosomal vesicles) [116], and NH_4_Cl, the intracellular amount of CSFV decreased. V-ATPase knockdown significantly reduced viral RNA copies, E2 protein levels, and CSFV titers. The conclusion was that CSFV entry into 3D4/21 cells is pH-dependent. MβCD pretreatment also led to reduced viral infection, indicating that cellular membrane cholesterol is also involved in clathrin-independent CSFV entry [109]. Taken together, these data clarify that the endocytosis pathway utilized by CSFV is cell-specific. CSFV entry into 3D4/21 cells is not dependent on clathrin but on caveolae.

## 5. Intracellular Trafficking

After entering into the host cells via internalization pathways mediated by clathrin or caveolae, most viruses utilize the endosomal system, including early endosomes (EEs), late endosomes (LEs), and lysosomes, to facilitate intracellular viral transmission. During this period, the cytoskeleton, including microfilaments and microtubules, provides transportation, and members of the small GTPase family act as regulators of intracellular viral trafficking. During this process, membrane fusion takes place, the viral nucleocapsid and RNA are released upon the promotion of some members of the endosomal sorting complex required for transport (ESCRT) family, and then transfer to the Golgi for protein processing and assembly. Viruses can also facilitate particle assembly and production by regulating ER–Golgi anterograde and retrograde trafficking, followed by extracellular budding of viral particles (Figure 2).

### 5.1. Endosomal Trafficking of CSFV from Endosomes to Lysosomes

The step from early to late endosomes is essential for the selective transportation of cargo and membrane components to lysosomes for degradation. The small GTPase Rab proteins (Rab5, Rab7, Rab7, Rab9, Rab11, etc.) regulate this process [140,141]. Among these proteins, Rab5 and Rab7 are involved in coordinating the transition from early to late endosomes [142,143]. Rab9 participates in the migration of cargo between late endosomes and the trans-Golgi network [144,145,146]. Rab11 is involved in perinuclear recycling endosomes and the trans-Golgi network [144,147,148].

The overexpression of DN mutant Rab5 S34N and mutant Rab7 T22N, but not mutant Rab11 S25N, significantly decreased CSFV replication in PK-15 cells. The knockdown of Rab5 or Rab7 caused a similar inhibitory effect on viral infection. Confocal microscopy showed that virus-containing vesicles acquired Rab5 endosomes after 30 min of internalization and transitioned to Rab7 endosomes at 45 mpi [108]. In 3D4/21 cells, in addition to Rab5 and Rab7, Rab11 was involved as a regulator of vesicular trafficking to recycling endosomes. Overexpression of Rab5 S34N, Rab7 T22N, or Rab11 S25N restrained CSFV infection, whereas DN mutant Rab9 Q66L exerted no inhibitory effect on CSFV. The knockdown of Rab5, Rab7, Rab11, or lysosomal-associated membrane protein 1 (LAMP-1) by siRNA interference reduced viral RNA copy numbers and virus titers, while si-Rab9 had no effect. CSFV virions colocalized with Rab5, Rab7, Rab11, and LAMP-1, but not with Rab9, at 30 mpi. This indicates that Rab5, Rab7, Rab11, and LAMP-1 guide virions through the transport route from early to late and even recycling endosomes, followed by a transfer to lysosomes before viral RNA release [109].

Wang et al. reported for the first time that Rab22a also has a crucial function in CSFV entry into PK-15 cells by causing a Rab22a–Rab5–NS4B cascade. CSFV enhanced Rab22a expression in vivo and in vitro, as detected in piglet tissues (spleen, tonsil, and intestine) and PK-15 cells. Rab22a overexpression increased the viral genome copies and CSFV E2 expression and its knockdown suppressed viral production. As Rab22a acts as a molecular switch of the GTP-bound form [149], the mutants Rab22a Q64L and Rab22a S19N, which lock Rab22a in the GTP-bound and GDP-bound forms, respectively, were transfected into cells. By disrupting Rab22a-mediated vesicular transport [150], CSFV infection was impeded. Moreover, CSFV NS4B interacted with Rab22a and colocalized with Rab5, forming a triple complex in early endosomes in PK-15 cells, which may facilitate viral entry [151].

COP I, which is located on endosomes, is a major substrate of ADP-ribosylation factors (ARFs, which are small GTPases of the Ras family) and coordinates the transition of cellular composition through vesicles in the early secretory pathway. COP I and ARF1 form a complex with Golgi-specific brefeldin A resistance factor 1 (GBF1) [152]. Zhang et al. first demonstrated that the GBF1–ARF1–COP I axis mediates CSFV transportation from early to late endosomes, promoting viral entry into swine umbilical vein endothelial cells (SUVECs) [153]. GBF1 was previously validated as being important in CSFV replication in SUVECs [154].

To further determine the functional domains of GBF1 in CSFV infection, a series of GBF1 truncation mutants were generated, among which only the Sec7 domain-containing plasmid restored the level of CSFV replication upon Golgi-specific brefeldin A (BFA, which is a GBF1 inhibitor) or golgicide A (GCA, which is a GBF1 Sec7 domain inhibitor) treatment. Moreover, CSFV replication was dampened, and viral plaques were fewer and smaller in COP I subunit KO cell lines. COP I was also found to be dispensable in CSFV binding and entry. The CSFV genome copy numbers were reduced in COP-KO cell lines and BFA- and GCA-treated cells according to RT-qPCR. CSFV particles were observed to colocalize with Rab5 and Rab7 in SUVECs, just like in PK-15 and 3D4/21 cells [108,109]. When SUVECs were pretreated with the BFA and GCA inhibitors, confocal microscopy showed a decrease in the number of CSFV particles colocalized with Rab7, but not Rab5, demonstrating that the trafficking of CSFV particles to late endosomes was defective. Furthermore, colocalization between COP I subunit COP β and CSFV C/E2 protein was detected. Meanwhile, CSFV infection promoted GBF1, ARF, and COP I mRNA expression. These results indicate that CSFV infection induces the expression of the GBF1–ARF–COP I complex and mediates the distribution of COP I-coated vesicles [153].

ESCRT, which is pivotal molecular machinery for the endosomal/multivesicular body (MVB) protein-sorting pathway in eukaryotic cells; regulates membrane invagination during endocytosis; participates in vesicle formation; and sorts newly synthesized membrane proteins from trans-Golgi vesicles into the endosomes, lysosomes, or plasma membranes [155,156]. ESCRT can also be responsible for the intracellular virus life cycle, including entry and budding [157]. Using confocal microscopy, Liu et al. discovered that the ESCRT-I complex Tsg101 protein assisted in the clathrin-mediated endocytosis of CSFV, delivering the virus from the late endosome (Rab7 and Rab9) to the lysosome (LAMP-1). Following uncoating, transit to the ER occurred. Subsequently, Tsg101 was found to interact with CSFV NS4B and NS5B and was involved in the formation of a viral replication complex (VRC), thereby regulating CSFV genome replication [158].

Furthermore, systematic siRNA screening was employed to identify the endogenous ESCRT subunits that participate in CSFV infection. VPS25 (a subunit of ESCRT-II), along with CHMP4B and CHMP7 (subunits of ESCRT-III), were recruited to the entry site of CSFV particles with clathrin and different endosomes, but VPS25 merged with clathrin only. The interactions between viral proteins and ESCRT subunits were examined using confocal fluorescence microscopy and immunoprecipitation. HRS (a subunit of ESCRT-0) colocalized with NS5A, VPS28 (a subunit of ESCRT-I) colocalized with NS4B, VPS25 colocalized with NS3/NS4B, VPS4 (a component of an independent complex from ESCRT-0 to ESCRT-III) colocalized with NS5A/NS5B, and ALIX (combined protein with ESCRT-I and ESCRT-III) colocalized with NS4B, while CHMP2B (a subunit of ESCRT-III), CHMP4B, and CHMP7 interacted with NS4B, NS3/NS4B, and NS5A, respectively. In addition, these ESCRT subunits were colocalized with double-stranded RNA (dsRNA), which is an intermediate product of CSFV genome replication, and they were all located in the ER but not the Golgi, helping to create a specific replication complex for efficient viral genome replication. A new schematic regarding ESCRT subunits in the CSFV life cycle was systematically provided showing that Tsg101, VPS25, CHMP4B, and CHMP7 interacted with clathrin upon CSFV entry, whereas CHMP4B and CHMP7 were localized with the Rab5 protein in the early endosomes, and CSFV transferred to the late endosomes with Tsg101, CHMP4B, CHMP7, and Rab9 proteins. After CSFV was relayed to the lysosomes, the Tsg101 and CHMP4B proteins were associated with LAMP-1, leading to uncoating and nucleic acid release, followed by transportation to the ER area [159,160].

The role of valosin-containing protein (VCP/p97) in CSFV intracellular transport was recently described by Sun et al. VCP is a member of the type II AAA^+^ ATPase family and is mainly distributed in the cytoplasm, with a fraction associated with subcellular organelle membranes [161]. It was shown to be involved in the life cycle of many viruses [162,163,164,165,166]. Treatment with VCP inhibitors DBeQ, NMS-873, and ML-240 impaired CSFV propagation in PK-15 cells. VCP knockdown inhibited and overexpression promoted CSFV replication according to viral mRNA and protein levels assessed using RT-qPCR, Western blotting, and an immunofluorescence assay. VCP affected viral entry but not viral binding such that viral gene copy numbers in VCP-knockdown cells showed no change at 0 hpi but declined significantly at 1, 2, and 6 hpi. VCP showed enhanced protein levels within 90 min post-infection, and obviously colocalized with CSFV particles. In IF and IP experiments, VCP interacted with Rab5 and LAMP-1, but not with clathrin, Rab7, Rab9, or Rab11, and its silencing arrested virus accumulation in Rab5 early endosomes instead of transmission to Rab7. This demonstrates that VCP regulates intracellular trafficking of CSFV by participating in early endosome maturation and promoting CSFV release from early endosomes [167].

### 5.2. Trafficking of CSFV Mediated by Microfilaments and Microtubules

The cytoskeleton network plays a role in viral infection. For example, it acts as a scaffold in the perinuclear replication complex and cell cortex, and it can also act as a railway connecting early to late endosome transportation and late endosome to lysosome degradation [168]. Viruses usually invade host cells by promoting cytoskeleton remodeling [169,170,171]. The cytoskeleton is composed of actin filaments, microtubules, and intermediate filaments [172]. An actin cytoskeleton is a dynamically assembled structure, the polymerization of which was shown to participate in clathrin-mediated endocytosis by coupling with the plasma membrane and providing an invaginated pathway for viral internalization [173,174,175]. Microtubules are believed to be crucial in intracellular viral trafficking, assisting in shuttling the virus through the crowded cytoplasm to achieve effective replication [176,177].

In PK-15 and 3D4/21 cells, F-actin stress fibers were found to be evenly distributed in mock-infected cells but dissolved and accumulated when cells were infected with CSFV for 10 min. Later, at 1 hpi, stress fiber dissolution and accumulation were aggravated, resulting in the formation of lamellipodia and filopodia. The F-actin inhibitors latrunculin A (Lat A), jasplakinolide (Jasp), and cytochalasin D (Cyto D) have an inhibitory effect on the entry and subsequent replication of CSFV by destroying the rearrangement of F-actin but do not influence viral binding. Pharmaceutical experiments and RNA interference assays showed that the EGFR–PI3K/MAPK–RhoA/Rac1/Cdc42–cofilin signaling pathways cause dynamic actin changes and regulate CSFV entry. Superimposed fluorescent spots of clathrin, F-actin, and virions were observed in PK-15 cells as early as 15 mpi, which intensified at 30 mpi and was impaired at 60 mpi, indicating that most particles were driven by clathrin–actin filaments to the early endosomes after their uptake. Rab5, CSFV particles, and F-actin with lamellipodia were colocalized at 30 mpi, and clear colocalization among Rab5, virions, and F-actin with dissolved stress fibers was detected at 90 mpi. At 4 hpi, colocalization was reduced, suggesting that later transport of EEs no longer requires the assistance of F-actin [178].

To determine the role of microtubules in CSFV trafficking, PK-15 cells were treated with nocodazole (a microtubule inhibitor) at different time points of CSFV inoculation. Drug treatment 1 h before CSFV infection or 3 hpi significantly inhibited viral infection, while post-treatment within 1 hpi had no obvious influence. Furthermore, confocal microscopy analysis showed colocalization of tubulin, virions, and Rab7, as well as tubulin, virions, and LAMP-1. These results indicate that microtubules regulate Rab7-mediated late endosome and LAMP-1-mediated lysosome transportation in the life cycle of CSFV. The bidirectional delivery of cargo through microtubules relies on molecular motors: minus-end motors (dynein) and plus-end motors (kinesin) [179,180]. Chemical pretreatment with ciliobrevin D (a specific inhibitor of dynein) and RNA interference targeting dynein reduced CSFV RNA copy numbers. In addition, microscopy analysis showed evident colocalization of virions, dynein, and microtubules [178].

Recently, the positive role of kinesin-1 in CSFV endocytic trafficking along acetylated microtubules was reported. Knockdown and overexpression of kinesin-1 heavy chain (Kif5B) suppressed and promoted CSFV proliferation, respectively, at indicated time points post-infection. The colocalization of Kif5B and Rab7, Rab11, or LAMP-1 reflected its association with endosomal and lysosomal trafficking of CSFV in the early stage, and the interaction between Kif5B and dynein revealed their cooperation in guiding the bidirectional movement of CSFV particles along microtubules. A new schematic was proposed indicating that kinesin-1 and dynein are synergistically involved in the bidirectional transport of CSFV, during which dynein loads late, recycling endosomes or lysosomes with encapsulated CSFV particles, while kinesin-1 takes charge when bypassing obstacles. The intermediate filaments are extensible, which supports cellular plasticity and protects cells from outside mechanical forces [181]. Vimentin is one of the pivotal proteins in the formation of intermediate filaments [182]. As vimentin showed no colocalization with virions, and as viral RNA copies were not reduced when vimentin was knocked down, it was considered to be dispensable in CSFV intracellular transport [183].

## 6. Conclusions

Viral attachment, internalization, and trafficking are early events of CSFV infection and are indispensable for virus proliferation. This review focused on the relevant findings regarding the stages of the CSFV life cycle. Here, we summarized the identified and putative factors exploited for CSFV attachment and endocytosis, including HS, LamR, CD46, MERTK, and ADAM17 (Table 1). However, eliminating any one of them does not entirely eradicate viral infection. How these proteins specifically function in CSFV invasion and whether yet unknown additional factors are required need further verification. CSFV enters host cells through the internalization pathway, which is dependent on clathrin or caveolae, and then proceeds through the process of intracellular trafficking. Virions are further transported intracellularly through the transition from early to late endosomes, where actin filaments and the microtubule cytoskeleton are involved (Figure 2). Although much work has been undertaken, the details of these intricate mechanisms remain elusive. Several questions have been raised. For example, as the endocytosis pathway of CSFV differs in PK-15 and 3D4/21 cells, which pathway is employed in other cell lines and what exactly is the internalization mechanism in vivo remain to be elucidated. How capsids engage particular molecular motors, including cytosolic dynein and kinesin, in anterograde and retrograde transport, awaits to be addressed. The interaction between viral and host cell proteins and the functions of distinct proteins should be further explored. A better understanding of the biology of the virus can contribute to the development of CSF control, and further exploration of drugs and/or small molecules that can simultaneously target receptors and signal molecules could provide insights regarding antiviral strategies.

## Figures and Tables

**Table 1:** Involving entry factors of CSFV.

Protein Name	Interaction with Viral Proteins	Function	Reference
HS	E^rns^	Receptor	[17,18]
LamR	E^rns^	Receptor	[19]
CD46	**-**	Receptor	[20,67]
MERTK	E2	Attachment factor	[64]
ADAM17	E2	Attachment factor	[65]
Anx2	E2	Attachment factor	[68]
LDLR	**-**	Attachment factor	[19]

“-” in the table means that no experiments have shown this protein binds to any viral protein.

## Data Availability

Data sharing is not applicable to this article as no new data were created or analyzed in this study.
